# Metagenomic next-generation sequencing for the diagnosis of invasive pulmonary *aspergillosis* in type 2 diabetes mellitus patients

**DOI:** 10.1038/s41598-024-67174-8

**Published:** 2024-07-18

**Authors:** Zhiyun Liu, Hengxin Chen, Dubo Chen, Xianjin Wu, Hongxu Xu, Peisong Chen, Ruizhi Wang, Yili Chen

**Affiliations:** 1 Department of Laboratory Medicine, The First Affiliated Hospital of Sun Yat-Sen University, Guangzhou, 510080 Guangdong China; 2 Department of Clinical Laboratory, Huizhou Central People’s Hospital, Huizhou, 516001 Guangdong China

**Keywords:** Metagenomic next-generation sequencing, Invasive pulmonary *aspergillosis*, Type 2 diabetes mellitus, Diagnosis, Biological techniques, Microbiology, Molecular biology

## Abstract

Invasive pulmonary aspergillosis (IPA) in patients with diabetes mellitus has high incidence, especially in Type 2 diabetes mellitus (T2DM). The aim of this study was to evaluate the diagnostic efficacy of metagenomic next-generation sequencing (mNGS) for IPA in patients with T2DM. A total of 66 patients with T2DM were included, including 21 IPA and 45 non-IPA patients, from January 2022 to December 2022. The demographic characteristics, comorbidities, laboratory test results, antibiotic treatment response, and 30-day mortality rate of patients were analyzed. The diagnostic accuracy of mNGS and conventional methods was compared, including sensitivity, specificity, positive predictive value and negative predictive value. The sensitivity and specificity of mNGS were 66.7% and 100.0%, respectively, which were significantly higher than those of fluorescence staining (42.1% and 100%), serum 1,3-β-D-glucan detection (38.1% and 90.9%), serum galactomannan detection (14.3% and 94.9%) and BALF galactomannan detection (47.3% and 70.7%). Although the sensitivity of BALF culture (75.0%) was higher than that of mNGS (66.7%), the turnover time of mNGS was significantly shorter than that of traditional culture (1.6 days vs. 5.0 days). The sensitivity of mNGS combined with BALF culture reached 100.0%. In addition, mNGS has a stronger ability to detect co-pathogens with IPA. 47.6% of T2DM patients with IPA were adjusted the initial antimicrobial therapy according to the mNGS results. This is the first study to focus on the diagnostic performance of mNGS in IPA infection in T2DM patients. MNGS can be used as a supplement to conventional methods for the diagnosis of IPA in patients with T2DM.

## Introduction

According to IDF Diabetes Atlas, 537 million adults (aged 20–79 years) will have diabetes in 2021. By 2030, this number is expected to reach 643 million, and by 2045 it will be up to 783 million, mainly type 2 diabetes (T2DM)^1,2^. Diabetes increases the risk of infection, and the prevalence of diabetes in patients with invasive *aspergillosis* is 17.5 – 30.9%^3,4^. Invasive pulmonary *aspergillosis* (IPA) is the most serious type of *aspergillosis*-related infection, with the worst prognosis and high mortality^5,6^. Delayed diagnosis and treatment is a major cause of high mortality in IPA^7^. Therefore, the early diagnosis of IPA in T2DM patients is particularly important to improve the prognosis.

At present, IPA still faces difficulties in early diagnosis and treatment^8^. In clinical practice, IPA has similar clinical symptoms with other lung diseases such as tuberculosis, including fever, chest tightness, shortness of breath, cough, chest pain, bloody sputum, weight loss, night sweats and so on^9^. The current diagnostic reference criteria include positive culture and/or histological results, but the sensitivity is relatively low. Conventional cultivation takes a long time and has a low detection rate^10^.

In recent years, non-invasive biomarkers such as serum 1,3-β-glucan (BDG), serum and bronchoalveolar lavage fluid (BALF) galactomannan (GM) have been widely used in the early detection of IPA. However, these test results are susceptible to interference from other factors, resulting in false positives or false negatives.. Different cutoff values need to be used for diagnosis of different populations, such as patients with granulocytopenia and non granulocytopenia. Generally, the sensitivity of BDG test was 80–90%, and the specificity was 36–92%. The sensitivity of serum GM test was 30–100%, and the specificity was 38–98%^11,12^. PCR is a method recommended by EORTC/MSGERC for the detection of *Aspergillus*, but studies have shown that the sensitivity and specificity of *Aspergillus* PCR analysis methods from different manufacturers are quite different. The sensitivity of Artus *Aspergillus* RG PCR was only 47.6%, while the sensitivity of MycAssay Aspergillus PCR was 61.9%^13^. Other studies have shown that the sensitivity of GM and Aspergillus PCR in blood is lower, only 31% and 0%^14^. The limitation and incomplete coverage of primer design may cause false negative results and delay diagnosis. The exact role of PCR in the diagnosis of IPA remains controversial^15^. There is an urgent need to explore faster and more accurate diagnostic tools for T2DM patients with IPA to identify IPA early, improve patient prognosis and reduce mortality.

Metagenomics next-generation sequencing (mNGS) is an unbiased pathogen detection and nucleic acid molecular sequencing technology with the advantages of high throughput and single detection. It is considered to be a promising microbial identification technology^16^. At present, mNGS has been widely used in the diagnosis of IPA. Yang et al. observed that the sensitivity of mNGS (lung biopsy, BALF) in the diagnosis of pulmonary fungal infection was significantly higher than that of conventional detection^17^. Another study showed that the diagnostic rate of mNGS for mixed pulmonary infection was 4 times higher than that of conventional methods^18^. Invasive *aspergillosis* is still a major invasive fungal infection with serious clinical consequences in immunocompromised patients^19^. However, the diagnostic performance of mNGS for IPA through BALF and blood samples of T2DM patients has rarely been reported.

In our study, we evaluated the performance of mNGS in BALF and blood samples in the diagnosis of IPA in T2DM patients.

## Methods

### Study participants

**Figure 1:**
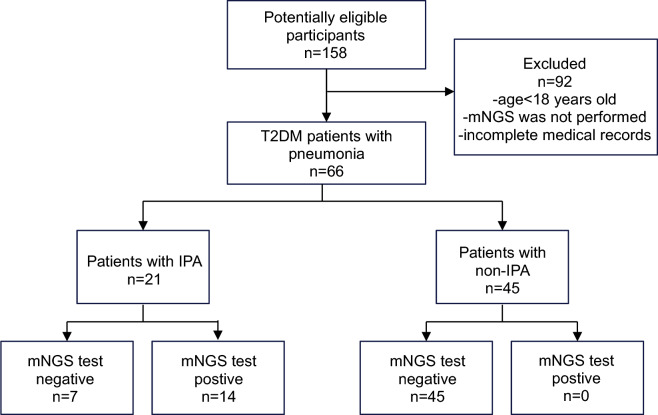
Flow chart of literature search.

This study was a retrospective study, and 66 patients with T2DM complicated with pneumonia who were hospitalized in the First Affiliated Hospital of Sun Yat-sen University from January 2022 to December 2022 were continuously included. Invasive fungal diseases (IFDs) were defined according to the consensus of the European Organization for Research and Treatment of Cancer and the Fungal Research Group Education and Research Consortium (EORTC/MSGERC)^20^. Clinical diagnosis of IPA or non-IPA was made by two senior pulmonary experts after discussion with the medical team based on clinical symptoms, laboratory tests, chest imaging, microbiological examination and treatment response. Finally, 21 IPA patients and 45 non-IPA patients were diagnosed. Exclusion criteria : (1) age < 18 years; (2) No mNGS; (3) Incomplete medical records (Fig. 1). The study was approved by the First Affiliated Hospital of Sun Yat-sen University and in accordance with the Helsinki Declaration.

### Sample collection and etiological diagnosis

BALF was collected according to the guidelines^21^. After contraindications were excluded, all patients underwent bronchoscopy under intravenous anesthesia or 2% lidocaine surface anesthesia. After the same amount of normal saline was injected into the bronchial segment of the affected side, the negative pressure suction BALF was used for related detection.

At the same time, BALF mNGS and conventional methods were used to detect the etiology of BALF and peripheral blood in all patients. The microbiological detection of IPA in this study included serum BDG, GM test and mNGS. Other etiological laboratory diagnoses also include traditional culture methods and immunofluorescence staining. BDG was detected according to the method recommended by the manufacturer (Dana Biotechnology Co., Ltd., China). Sandwich immune capture enzyme-linked immunosorbent assay (Platelia Aspergillus Ag; bio-Rad, Marnes-la-Coket, France) was used to detect galactomannan antigen in serum and BAL fluid. Immunofluorescence staining was performed using a fungal fluorescent staining solution (Nanjing Hanrui Biotechnology Co., Ltd., China).

### Sample processing and DNA extraction for mNGS

BALF 600 μL of each patient was taken and mixed with lysozyme and glass beads. Then, the mixture was placed in a horizontal platform of a vortex mixer and stirred violently at 2800–3200 rpm for 30 min. For nucleic acid extraction from BALF, we transferred 200 μL of the supernatant into a 2 mL centrifuge tube. The patient 's blood (3–5 mL) was centrifuged at 4 °C and 3500 rpm for 10 min to separate plasma. To extract DNA from the blood, we transferred 200 μL of plasma into a 2 mL sterile tube. DNA was then extracted using the IDseq TM Micro DNA Kit (Visual Medicine, VM002-50D, China) according to standard procedures^22^.

### Library preparation and sequencing construction

DNA library was constructed by transposase-based method. After purification and size selection, the concentration of the library was determined using a Qubit instrument before loading. The mixed library was sequenced on the Illumina Next Seq 550 system using a 75 bp single-end sequencing kit. The qualified result was that no less than 10 million reads were obtained for each sample, and the Q30 score was ≥ 85%. During each sequencing, the negative control samples were subjected to parallel processing and sequencing for quality control^23^.

### Bioinformatic analysis

Fastp software^24^ was used to remove low-quality and short (length < 35bp) reads to generate high-quality sequencing data. The human host sequence is reduced by using the Burrows-Wheeler paper delivery device tool (BWA) to map to the human reference genome sequence (assembled by the National Biotechnology Information Center GRCh38)^25^. After removing low-complexity reads, the remaining data were classified by comparing them to the constructed microbial genome database of viruses, bacteria, fungi and parasites. We developed a set of criteria similar to the National Center for Biotechnology Information (NCBI) (https://www.ncbi.nlm.nih.gov/ genome /) to select representative combinations of microorganisms.After quantifying each microorganism, it is very important to remove the contamination in the reagent. In order to determine the list of background microorganisms, we classified the microorganisms detected in at least 25% of the samples, including negative controls. The correlation between the quantitative value of each microbial abundance and the total amount of nucleic acid was tested. When the quantitative value of the abundance of the microorganism is negatively correlated with the total amount of nucleic acid in the sample, it is determined to be a reagent-derived background organism. Therefore, they are excluded from the report.The sequencing data list was analyzed according to the number of strictly aligned reads (SMRN, representing species-specific sequences), genome coverage (%), and relative abundance (%).

### Threshold criteria for interpretation of metagenomic analysis

The microbial list obtained from the above analysis process was compared with an internal background database, which contained microorganisms that appeared in more than 50% of the samples in the laboratory in the past three months. Remove suspected background microorganisms from the list of microorganisms. For different types of microorganisms, the threshold is set as follows : (1) Extracellular bacteria/fungi (excluding Cryptococcus)/parasites : SMRN ≥ 30, ranking the top 10 in bacteria, fungi or parasites. The organisms detected in the negative control group or ≥ 25% of the samples in the first 30 days were excluded, but only if the detected SMRN was ≥ 10 times that of the negative control group or other organisms. In addition, microorganisms present in ≥ 75% of the samples within the first 30 days were excluded. (2) Intracellular bacteria (excluding Mycobacterium tuberculosis and Brucella)/Cryptococcus : SMRN ≥ 10, the top 10 were bacteria or fungi. Pathogens detected in the negative control group or in ≥ 25% of the samples in the first 30 days were excluded, but only when the detected SMRN was ≥ 10 times that of the negative control group or other organisms. (3) Virus/Brucella : SMRN ≥ 3, the pathogens detected in the negative control group were excluded, but only when the detected SMRN was more than 10 times that of the negative control group. (4) Mycobacterium tuberculosis : SMRN ≥ 1^22,26^. The way to distinguish true pathogens from contaminants is through the frequency of specific bacteria, the "intra-genus ranking third and later", the "NC filter" and the alignment results of a pathogen. What more, the thorough decision on pathogenic bacteria must also be made based on batch testing and patient clinical data.

### Clinical data collection

The clinical parameters of each patient were obtained by consulting the electronic medical records. The clinical features, underlying diseases, laboratory tests, imaging features, immunosuppressant use, antibiotic treatment response and 30-day mortality

### Statistical analysis

Statistical analysis was performed using online statistical tools (http://dxonline.deepwise.com/) and Graphpad prism.Continuous variables are expressed as mean ± standard deviatio or medians and interquartile. Categorical variables are expressed as counts and percentages. Two independent samples t test was used to compare the normal distribution of continuous variables between IPA and non-IPA patients. Mann–Whitney U test was used to compare the non-normal distribution of continuous variables. Chi-square test and Fisher exact tests were used for categorical variables. The sensitivity, specificity, positive predictive value (PPV) and negative predictive value (NPV) of mNGS, conventional pathogenic methods, serum 1,3-β-D-glucan test (BDG test), galactomannan test (GM test) and immunofluorescence staining in the diagnosis of IPA in T2DM patients were calculated with the combined clinical diagnosis as the reference standard. Wilson 's method was used to calculate the 95% confidence interval of these ratios. *P* < 0.05 was considered statistically significant.

### Ethical approval

All methods were carried out in accordance with the declaration of Helsinki. Written informed consent was obtained from all subjects. This study was approved by the Clinical Research and Ethics Committee of the First Affiliated Hospital of Sun Yat-sen University.

## Result

### Clinical characteristics and laboratory findings

In this study, there were 66 patients with T2DM complicated with pneumonia, including 21 patients with IPA and 45 patients with non-IPA. The median age (69.0 vs. 69.2 years) and gender composition of the two groups were similar. The most common clinical symptoms of IPA in T2DM patients were fever (57.1%), cough (42.9%) and dyspnea (33.3%). Comparing the two results, sputum was observed less frequently in IPA patients. Among the underlying diseases of IPA patients, emphysema was more common than non-IPA group (*p* = 0.005).Both groups showed different degrees of immunosuppression. The use of broad-spectrum antibiotics (23.8%), glucocorticoids (23.8%), and cyclophosphamide (19.0%) ranked first among IPA patients. In contrast, immunosuppression was much less common in non-IPA patients.

In T2DM patients with IPA, the most common imaging features of chest CT were patchy shadows (57.1%), bilateral lung inflammation (52.4%), multiple lesions (47.6%) and pleural effusion (42.9%). Some showed typical features, such as cavity sign (4.8%). In the laboratory examination results, the PCT and GM tests in bronchoalveolar lavage fluid or blood of IPA patients were higher than those of non-IPA patients, especially the blood GM test was significantly increased in T2DM patients with IPA (*P* = 0.001). The 30-day mortality of T2DM patients with IPA was significantly higher than that of the control group (*P* = 0.000). The positive results of GM test (BAL) and the occurrence of emphysema are independent risk factors for T2DM combined with IPA. (Table 1).

**Table 1:** Clinical characteristics, laboratory findings, and radiologic features of IPA and non-IPA in T2DM patients on admission.

Characteristics (median[IQR] or n[%])	Non-Plumonary aspergilosis group (n = 45)	IPA group (n = 21)	Univariable P value	Multivariable *P* value
Male	23 (51.1%)	16 (76.2%)	0.054	
Age(years)	69.022 ± 12.655	69.238 ± 8.871	0.944	
Smoke history	17 (37.8%)	8 (38.1%)	0.980	
Mechanical ventilation	29 (64.4%)	16 (76.2%)	0.340	
Broad-spectrum antimicrobial drugs	7 (15.6%)	5 (23.8%)	0.640	
30 days-mortality	0(0.0%)	8 (38.1%)	0.000 **	
Specimen Type for mNGS			0.057	
Sputum	2 (4.4%)	0 (0.0%)		
BALF	43 (95.6%)	19 (90.5%)		
Blood	0 (0.0%)	2 (9.5%)		
Clinical symptoms				
Cough	22 (48.9%)	9 (42.9%)	0.647	
Expectoration	20 (44.4%)	6 (28.6%)	0.219	
Hemoptysis	1 (2.2%)	0 (0.0%)	1.000	
Stuffiness	6 (13.3%)	1 (4.8%)	0.532	
Dyspnea	13 (28.9%)	7 (33.3%)	0.714	
Fever	20 (44.4%)	12 (57.1%)	0.336	
Chest CT images				
Bronchiectasis	1 (2.2%)	3 (14.3%)	0.174	
Nodular shadow	13 (28.9%)	5 (23.8%)	0.666	
Patky or patchy shadows	35 (77.8%)	12 (57.1%)	0.085	
Cavity sign	1 (2.2%)	1 (4.8%)	1.000	
Pleural effussion	19 (42.2%)	9 (42.9%)	0.961	
Mediastinal lymphoma adenopathy	1 (2.2%)	0 (0.0%)	1.000	
Multiple lesions	18 (40.0%)	10 (47.6%)	0.560	
Inflammation in both lungs	32 (71.1%)	11 (52.4%)	0.137	
Underlying diseases				
Interstitial pneumonia	3 (6.7%)	4 (19.0%)	0.275	
Emphysema	2 (4.4%)	7 (33.3%)	0.005 **	0.04**
COPD	1 (2.2%)	2 (9.5%)	0.489	
SLE	1(2.2%)	0(0.0%)	1.000	
Innutrition	3 (6.7%)	0 (0.0%)	0.564	
Bacillary phthisis	2 (4.4%)	1 (4.8%)	1.000	
Kidney transplant	2 (4.4%)	1 (4.8%)	1.000	
Liver transplantation	1 (2.2%)	1 (4.8%)	1.000	
Hematological malignancy	1 (2.2%)	1 (4.8%)	1.000	
Solid tumours	4 (8.9%)	1 (4.8%)	0.928	
HIV infection	0 (0.0%)	1 (4.8%)	0.694	
Immunocompromised conditions				
Use of corticosteroids	4 (8.9%)	5 (23.8%)	0.208	
Mattimycohenl ester	0 (0.0%)	2 (9.5%)	0.183	
Cyclophosphamide	1 (2.2%)	4 (19.0%)	0.057	
Use of B cell immunosuppressants such as Bruton tyrosine kinase inhibitors	1 (2.2%)	0 (0.0%)	1.000	
History of neutropenia [neutrophils < 0.5109 / L (< 500 / mm3) were more than 10 d]	0 (0.0%)	1 (4.8%)	0.694	
Laboratory findings				
WBC(109/L)	11.75 ± 6.58	10.41 ± 5.40	0.420	
CRP	93.75(21.42–144.76)	65.39(17.73–99.97)	0.652	
PCT	0.36(0.15–2.14)	1.36(0.18–3.23)	0.162	
Serum BDG	37.50(37.50–59.38)	37.50(37.50–106.78)	0.140	
GM test(BAL)	0.50(0.40–1.00)	1.80(0.90–3.65)	0.002 **	0.002**
GM test(Blood)	0.300(0.200–0.400)	0.400(0.300–0.700)	0.075	

COPD chronic obstructive pulmonary disease, Serum BDG Serum (1,3)-β-D-glucan, GM galactomannan, CT computed tomography,CRP C-reactive protein, PCT procalcitonin, BALF bronchoalveolar lavage fluid.

### Comparison of diagnostic performance among mNGS, BALF culture, serum BDG, serum and BALF GM, and fluorescence staining in T2DM patients with IPA

**Figure 2:**
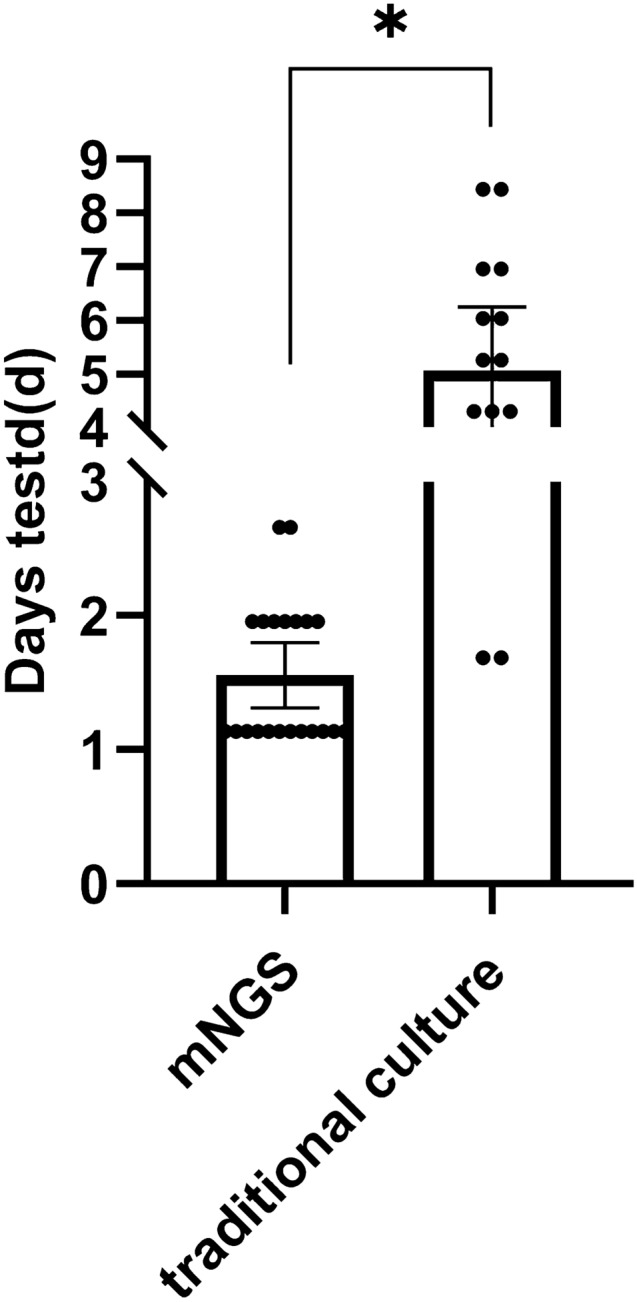
Comparison of results reporting time between mNGS and traditional culture.

The diagnostic accuracy of mNGS and BALF culture, fluorescence staining, serum BDG and serum and BALF GM test were compared. All patients ' BALF and / or serum were detected by mNGS. As shown in Table 2, the sensitivity and specificity of mNGS were 66.7% and 100.0%, respectively, which were significantly higher than those of fluorescence staining (FS) (42.1% and 100%), serum BDG (38.1% and 90.9%), serum GM (14.3% and 94.9%) and BALF GM (47.3% and 70.7%). Although the sensitivity of BALF culture (75.0%) seems to be higher than that of mNGS (66.7%) (Fig. 2), the TAT (turnaround time) of mNGS is much shorter than that of traditional culture (1.6 days vs. 5.0 days).

**Table 2:** Diagnostic performance of mNGS,BALF culture,serum BDG,serum GM, BALF GM and fluorescence staining in T2DM patients with IPA.

Methods	Plumonary aspergilosis cohort	Non-Plumonary aspergilosis cohort	Sensitivity (95% CI)	Specificity (95% CI)	PPV (95% CI)	NPV (95% CI)
mNGS + −	14 7	0 45	66.7%(0.431–0.845)	100.0%(0.902–1.000)	100.0%(0.732–1.000)	86.5%(0.736–0.940)
BALF culture + −	15 5	0 45	75.0%(0.505–0.904)	100.0%(0.902–1.000)	1.000(0.746–1.000)	90.0%(0.774–0.963)
Serum GM + −	3 18	2 37	14.3%(0.038–0.374)	94.9%(0.814–0.991)	60.0%(0.170–0.927)	67.2%(0.532–0.790)
BALF GM + −	9 10	12 29	47.37%(0.252–0.750)	70.7%(0.543–0.834)	42.6%(0.226–0.656)	74.4%(0.576–0.864)
Serum BDG + −	8 13	4 36	38.1%(0.190–0.613)	90.0%(0.754–0.967)	66.7%(0.354–0.887)	73.5%(0.587–0.850)
FS + −	8 11	0 41	42.1%(0.211–0.660)	100.0%(0.893–1.000)	100.0%(0.600–1.000)	78.8%(0.649–0.885)

FS fluorescence staining, serum BDG serum (1,3)-β-D-glucan, GM Galactose Mannitol, serum BDG > 100 ng/L was defifined as positive, GM > 1.0 ng/L was defifined as positive, CI confifidence intervals, PPV positive predict value, NPV negative predict value.

### Diagnostic performance of mNGS combine with conventional etiological methods

The diagnostic performance of mNGS combined with conventional pathogenic methods was analyzed. The sensitivity of mNGS combined with BALF culture reached 100.0%. When mNGS ran in parallel with conventional methods, the diagnostic performance was significantly improved (Table 3).

**Table 3:** Diagnostic performance of mNGS combine with conventional etiological methods.

Methods	Plumonary *aspergilosis* cohort	Non-Plumonary *aspergilosis* cohort	Sensitivity (95% CI)	Specificity (95% CI)	PPV (95% CI)	NPV (95% CI)
mNGS with BALF GM + -	19 2	12 29	90.5%(0.682–0.983)	70.7%(0.543–0.834)	61.3%(0.423–0.776)	93.5%(0.772–0.989)
mNGS with Serum BDG + -	16 5	4 36	76.2%(0.525–0.909)	90.0%(0.754–0.967)	80.0%(0.557–0.934)	87.8%(0.730–0.954)
mNGS with FS + -	16 5	0 41	76.2%(0.525–0.909)	100.0%(0.893–1.000)	100.0%(0.759–1.000)	89.1%(0.756–0.959)
mNGS with serum GM + -	14 7	2 37	66.7%(0.431–0.845)	94.9%(0.814–0.991)	87.5%(0.604–0.978)	84.1%(0.693–0.928)
mNGS with BALF culture + -	20 0	0 45	100.0%(0.800–1.000)	100.0%(0.902–1.000)	100.0%(0.800–1.000)	100.0%(0.902–1.000)

### The distribution of *Aspergillus* species detected by mNGS

**Figure 3:**
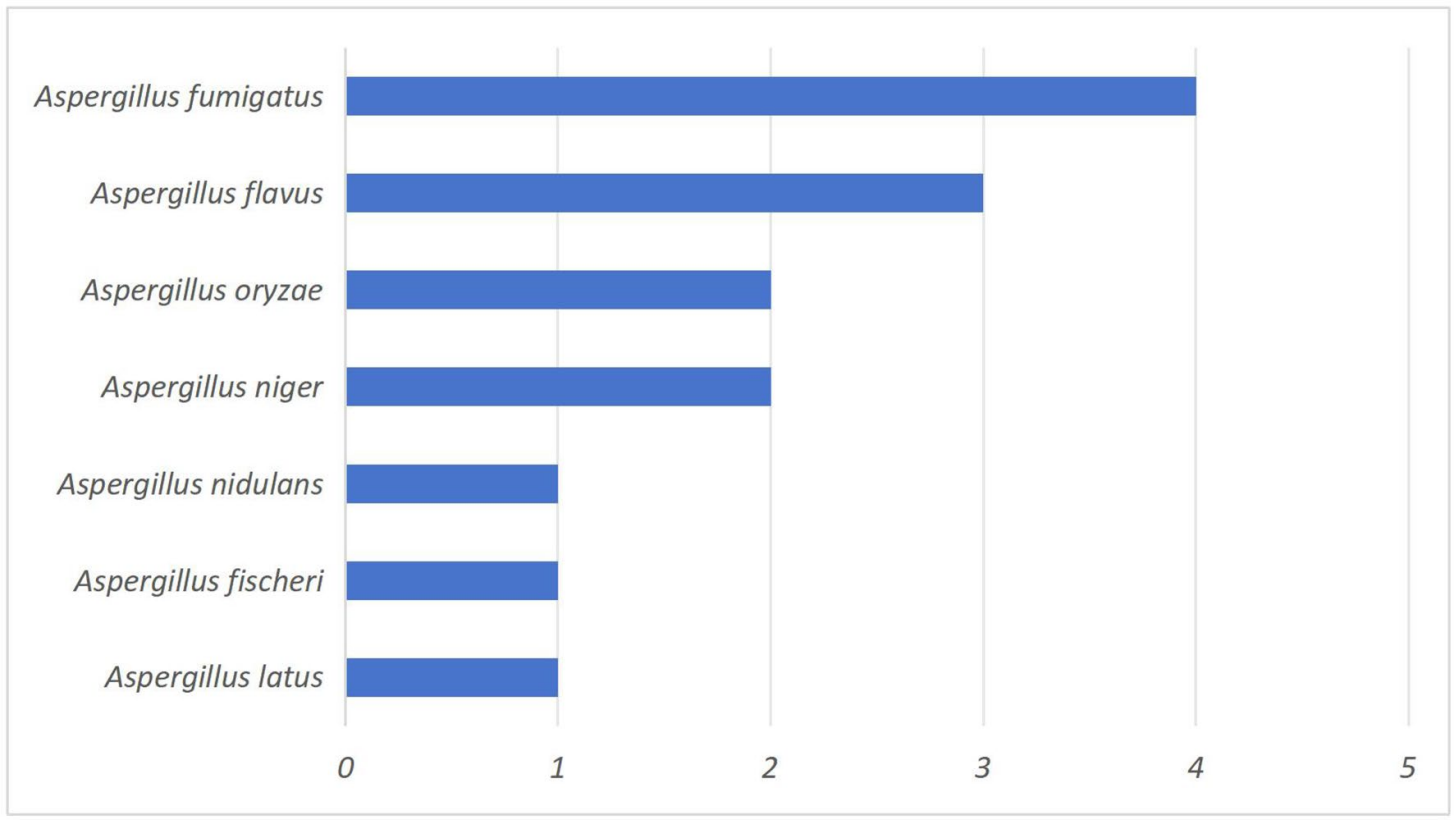
Distribution of Aspergillus species detected by mNGS.

Among the 21 patients with T2DM and IPA, 14 cases of *Aspergillus* were detected by mNGS, and the top two *Aspergillus* species were *Aspergillus fumigatus* and *Aspergillus flavus*(Fig. 3).

### Co-pathogens detected by mNGS

**Figure 4:**
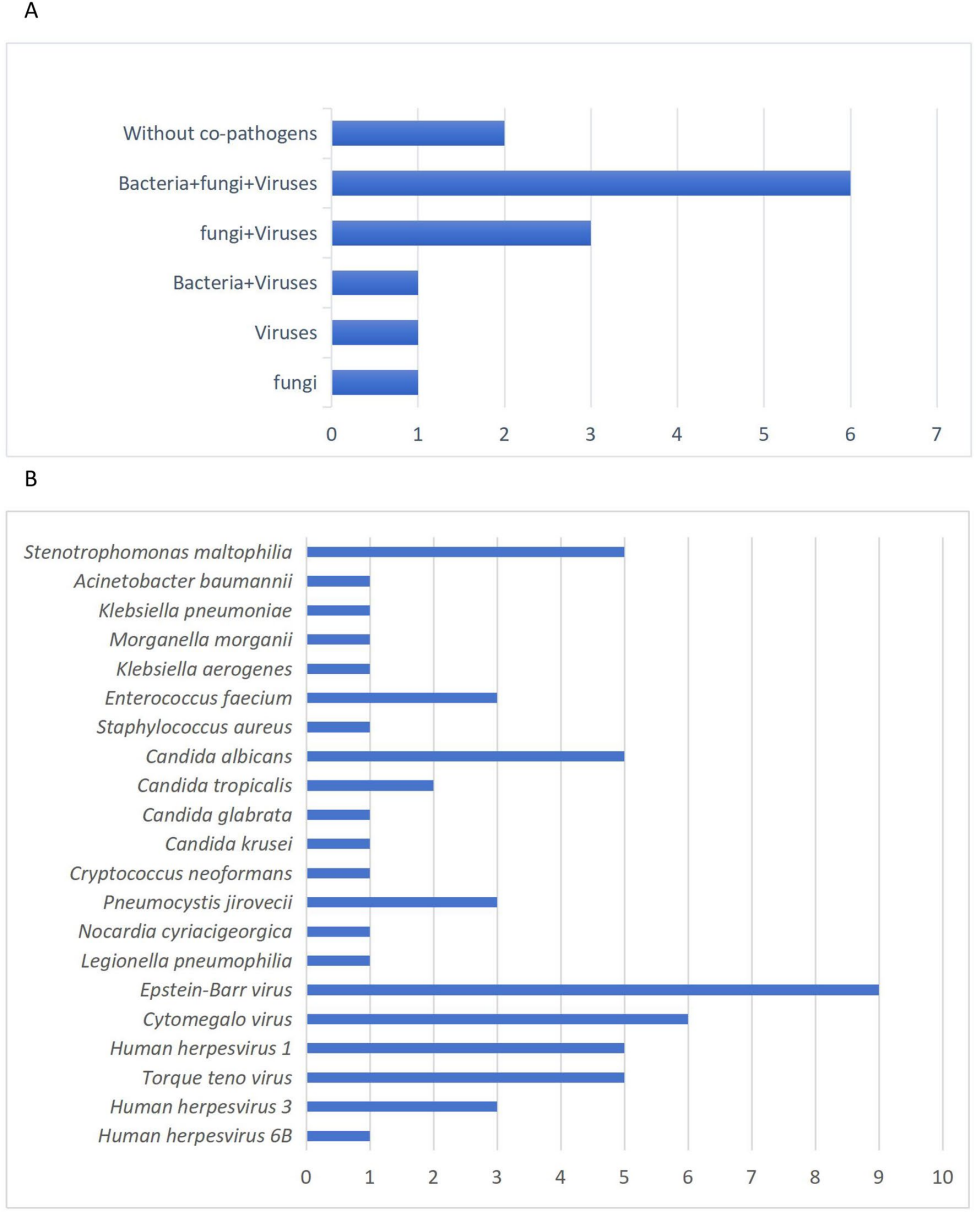
Co-pathogens identified by mNGS in 21 T2DM patients with IPA. (**A**) Number of IPA patients with co-pathogens identified by mNGS; (**B**) number of IPA patients infected with various co-pathogens.

Twelve cases of co-pathogen infection were diagnosed by mNGS (Fig. 4) . MNGS showed satisfactory performance in the identification of co-infected bacteria (12/14,85.7%). The most common co-infection pattern was Aspergillus-Bacteria-Fungi-Virus, a total of 6 cases (42.8%) (Fig. 4A). Only 14.2% of the patients in the observation group were only *Aspergillus* infection.The most common co-infected bacteria was *Stenotrophomonas maltophilia*, the most common fungus was *Candida albicans*, and the most common virus was *Epstein-Ban* virus. In addition, mNGS detected 3 cases of *Pneumocystis jiroveci* and 1 case of *Legionella pneumophila*, which was difficult to detect by conventional microbiological methods.

### Impact of mNGS on IPA patients’ antimicrobial therapy

According to mNGS, 47.6% of IPA patients modified the initial antimicrobial treatment regimen. 9.5% adjusted the antibacterial drugs or antibacterial spectrum, 4.7% began to use anti-aspergillus drugs, 14.3% began to use compound sulfamethoxazole (TMP-SMZ), and 47.6% began to use antiviral drugs (Table 4).

**Table 4:** Impact of mNGS on T2DM patients with IPA’antimicrobial therapy.

Modifications	T2DM patients with IPA(n = 21)
Increase antimicrobial spectrum	2(9.5%)
Add anti-Aspergillus drugs	2(9.5%)
Change anti-Aspergillus drugs	2(4.7%)
Add TMP-SMZ	2(9.5%)
Add anti-viral drugs	10(47.6%)

## Discussion

In the past few decades, the prevalence of DM has increased significantly in almost all countries, becoming a "growing epidemic", mainly due to the continuous increase in the incidence rate of T2DM^27^. The global prevalence of *aspergillosis* is estimated to reach 250,000 IA cases per year^28^. The risk of IPA in DM patients is 27% higher than that in the general population^29^. Given that IPA has the highest mortality rate and the worst prognosis, early, rapid, and accurate diagnosis is particularly important.

MNGS is an unbiased pathogen detection method, which has been increasingly used in infectious diseases in recent decades^30^. Many studies have evaluated the performance of mNGS in patients with different types of infections^31–34^. Zhang et al. found that in diabetic patients, the positive rate of pathogen mNGS in pulmonary infection cases was significantly higher than that of routine detection, which was mainly driven by the detection of dominant bacteria^35^. However, there is limited data on the application of metagenomic next-generation sequencing (mNGS) in the diagnosis of IPA in patients with T2DM. In our study, we evaluated the diagnostic ability of mNGS in T2DM with IPA, mainly focusing on the detection of *Aspergillus*, and briefly analyzed the detection of co-pathogens.

In this study, mNGS showed higher sensitivity than most conventional etiological methods. Similar to Lin et al.^30^, we found that the diagnostic sensitivity of BALF culture for IPA was slightly higher than that of mNGS. The results showed that it was difficult to identify *Aspergillus* fungi from thick polysaccharide cell wall by DNA/RNA extraction^36,37^, and the identification of *Aspergillus* fungi by mNGS was still a challenge. It is worth noting that mNGS technology cannot distinguish between colonized and infected pathogens, and mNGS of Aspergillus in BALF samples sometimes shows false positive results^38^. Although the BALF culture method has high sensitivity and specificity, the time required to report results is three times that of mNGS. Each single detection has obvious limitations, and combined detection can make up for the limitations of single detection to a certain extent. The diagnostic efficiency of combined detection was significantly improved, and the sensitivity and specificity of mNGS combined with BALF culture were increased to 100%.

Early and rapid microbiological diagnosis of pulmonary aspergillosis is a key step to achieve timely initiation of treatment and improve the prognosis of T2DM patients with IPA. Diabetes are considered immunocompromised due to negative effects of hyperglycemic environment that favors immune dysfunction such as damage to neutrophil function^39^. Compared with non-survivors, survivors had higher WBC counts (*P* < 0.05), suggesting that non-survivors may have innate immune dysfunction^40^. Because of impaired immune function, diabetic patients are susceptible to almost all pathogens^3^. Theoretically, mNGS can detect all known pathogens,including bacteria, fungi, viruses, mycobacterium tuberculosis, parasites and atypical pathogens, through a single experiment,while pathogen detected by conventional etiological methods is usually a single one,which shows that mNGS has more advantages in the diagnosis of co-pathogens^41,42^. In our study ,there were 3 cases of *Pneumocystis jirovecii* and 1 case of *Legionella pneumophilia* detected by mNGS,which were difficult to be detected by conventional detection methods.

There are few studies that focus on the prognosis of IPA in diabetic patients. It is reported that diabetes be considered as risk factors for invasive mould infections^43,44^. Sun et al. found that the prevalence of diabetes was 15.9% in the survival group and 24.4% in the death group (*P* = 0.04) in a study of invasive pulmonary tramulinosis^45^. In our research, there are four patients of the eight deceased patients were from the mNGS false-negative group. Four patients were all from ICU, with diseases and complications in other organs. Meanwhile, incomplete wall breaking of mNGS may lead to false negative results.

However, in the realm of routine clinical practice, mNGS is an expensive procedure and poses challenges for implementation in grassroots hospitals. Blind treatment based on mNGS alone is inappropriate. It is essential to rule out contamination by integrating clinical findings and results from conventional tests. MNGS has inherent limitations; it cannot differentiate between pathogenic colonization and active infection. Moreover, due to its heightened sensitivity, there is a risk of false positives, while the potential for incomplete cell wall disruption could result in false negatives. The interpretation of mNGS results must be approached with caution, especially when dealing with non-sterile sites and low sequence counts [46]. Furthermore, mNGS demands the expertise of skilled technical staff, a well-equipped laboratory, and adherence to standardized experimental protocols.

Our retrospective study has some limitations. First of all, as a retrospective study, the existence of selection bias and recall bias is inevitable. In addition, since PCR is not routinely carried out in our laboratory, the lack of some PCR methods fails to better evaluate the diagnostic value of mNGS, which will be further explored in future work. Furthermore, the sample size of this study was small and bias was unavoidable. In addition, due to the lack of consensus on the interpretation of mNGS results, it is difficult to determine whether the microorganisms reported by mNGS are important clinical pathogens or colonized microorganisms, which must be classified according to clinical characteristics, laboratory tests and parameters.

## Conclusion

MNGS had good diagnostic value, especially in pathogens,which were difficult to detect by conventional etiological methods. Combining conventional etiological methods can improve the diagnostic performance of mNGS in T2DM with IPA.

## Data Availability

The original contributions presented in the study are included in the article/Supplementary Material, further inquiries can be directed to the corresponding authors.
